# Predicting recurrent glioblastoma clinical outcome to immune checkpoint inhibition and low-dose bevacizumab with tumor in situ fluid circulating tumor DNA analysis

**DOI:** 10.1007/s00262-024-03774-7

**Published:** 2024-08-06

**Authors:** Guangzhong Guo, Ziyue Zhang, Jiubing Zhang, Dayang Wang, Sensen Xu, Guanzheng Liu, Yushuai Gao, Jie Mei, Zhaoyue Yan, Ruijiao Zhao, Meiyun Wang, Tianxiao Li, Xingyao Bu

**Affiliations:** 1 Department of Neurosurgery, Juha International Center for Neurosurgery, Glioma Clinical Diagnosis and Treatment Center of Henan Province, Glioma Engineering Research Center for Precision Diagnosis and Treatment of Henan Province, Zhengzhou University People’s Hospital, Zhengzhou, 450003 Henan China; 2 Henan Provincial Neurointerventional Engineering Research Center, Henan International Joint Laboratory of Cerebrovascular Disease, Henan Engineering Research Center of Cerebrovascular Intervention Innovation, Zhengzhou, Henan China; 3 Department of Cerebrovascular Disease, Zhengzhou University People’s Hospital, Henan Provincial People’s Hospital, Henan University People’s Hospital, Zhengzhou, Henan China; 4 Department of Pathology, Zhengzhou University People’s Hospital, Henan Provincial People’s Hospital, Henan University People’s Hospital, Zhengzhou, Henan China; 5 Department of Radiology, Zhengzhou University People’s Hospital, Henan Provincial People’s Hospital, Henan University People’s Hospital, Zhengzhou, Henan China

**Keywords:** Glioblastoma, Recurrence, Phase II trials, Circulating tumor DNA, Prognosis, Biomarkers

## Abstract

**Objective:**

Most recurrent glioblastoma (rGBM) patients do not benefit from immune checkpoint inhibition, emphasizing the necessity for response biomarkers. This study evaluates whether tumor in situ fluid (TISF) circulating tumor DNA (ctDNA) could serve as a biomarker for response to low-dose bevacizumab (Bev) plus anti-PD-1 therapy in rGBM patients, aiming to enhance systemic responses to immunotherapy.

**Methods:**

In this phase II trial, 32 GBM patients with first recurrence after standard therapy were enrolled and then received tislelizumab plus low-dose Bev each cycle. TISF samples were analyzed for ctDNA using a 551-gene panel before each treatment.

**Results:**

The median progression-free survival (mPFS) and overall survival (mOS) were 8.2 months (95% CI, 5.2–11.1) and 14.3 months (95% CI, 6.5–22.1), respectively. The 12-month OS was 43.8%, and the objective response rate was 56.3%. Patients with more than 20% reduction in the mutant allele fraction and tumor mutational burden after treatment were significantly associated with better prognosis compared to baseline TISF-ctDNA. Among detectable gene mutations, patients with MUC16 mutation, EGFR mutation & amplification, SRSF2 amplification, and H3F3B amplification were significantly associated with worse prognosis.

**Conclusions:**

Low-dose Bev plus anti-PD-1 therapy significantly improves OS in rGBM patients, offering guiding significance for future individualized treatment strategies. TISF-ctDNA can monitor rGBM patients' response to combination therapy and guide treatment.

**Clinical trial registration:**

This trial is registered with ClinicalTrials.gov, NCT05540275.

## Introduction

Glioblastoma (GBM) is the most malignant primary brain tumor and is prone to recurrence. Despite multidisciplinary treatments including surgery, radiotherapy, chemotherapy, targeted therapy, and supportive care, the overall prognosis remains poor [1–4]. Updated guidelines for the management of gliomas still encourage clinical trials for rGBM due to the limited efficacy of available salvage therapies at the time of tumor recurrence, with a median survival of only 6–8 month [5]. The search for novel therapeutic options to improve the prognosis of rGBM patients is ongoing, with research focusing on combining antiangiogenic agents with immunotherapy to enhance antitumor immune responses [6, 7].

Preliminary results have shown that immune checkpoint inhibitors combined with anti-angiogenesis drugs have a good safety profile in treating rGBM [8, 9]. Non-clinical studies have demonstrated that bevacizumab, an antiangiogenic targeted agent, can inhibit vascular endothelial growth factor, promote tumor vascular normalization, increase T cell infiltration, and reduce immunosuppressive cell activity, thereby improving immunotherapy efficacy [10]. Although bevacizumab combined with immunotherapy has been feasible and safe in treating other solid tumors, it has not improved OS of rGBM patients [11]. A complete response to concurrent anti-PD-1 and low-dose anti-VEGF therapy was reported in one patient with rGBM [12]. Therefore, larger clinical trials are needed to investigate whether low-dose Bev can promote immunotherapy responses.

Biomarkers are critical to maximizing therapeutic efficacy and minimizing toxicity in rGBM treated with low-dose Bev plus anti-PD-1 therapy [13–16]. Analyzing circulating tumor DNA (ctDNA) as an emerging biomarker in solid tumors faces technical challenges due to the specificity of GBM's location [17, 18]. The collection of TISF for ctDNA analysis has been reported by our research group multiple times, yet there is limited literature on ctDNA changes in rGBM after immunotherapy combined with low-dose Bev treatment [17–20]. Thus, the feasibility of ctDNA as a biomarker in rGBM patients needs further investigation.

Combining tislelizumab with low-dose Bev in treating rGBM, we hypothesized that low-dose Bev treatment might improve the immunotherapy response. To assess ctDNA's efficacy for monitoring rGBM patients' response to combination therapy, we collected TISF samples at baseline and each subsequent immunotherapy cycle.

## Methods

### Study design and participants

This open-label phase 2 study (Clinical Trials ID: NCT 05540275) recruited rGBM patients at Zhengzhou University People's Hospital (Zhengzhou University). From March 28, 2022, patients received tislelizumab (200 mg) and bevacizumab (3 mg/kg) intravenously every 3 weeks until disease progression or intolerance. Magnetic resonance imaging (MRI) was performed at baseline and every 4–8 weeks thereafter. Tumor volume measurement and RANO 2.0 assessment were performed using 3D slicer software (National Institutes of Health, Bethesda, USA) [21].

Eligible patients were aged 18–75 years with confirmed rGBM, a Karnofsky Performance Status (KPS) ≥ 70, and had undergone ≥ 1 prior systemic GBM therapy. Exclusion criteria included systemic glucocorticoid or other immunosuppressive therapy within 7 days after enrollment, known or suspected active autoimmune disease, active hepatitis B or C, HIV infection, extracranial metastases, significant leptomeningeal disease, or tumors primarily in the brain stem or spinal cord.

### Treatment Regimens

Primary GBM: Patients received concurrent chemoradiotherapy (TMZ 75 mg/m^2^/d for 42 days) 4 weeks after surgery, followed by TMZ (150 mg/m^2^/d every 4 weeks for 5 days, repeated every 28 days for 6 cycles).

Recurrent GBM: Surgery was recommended. Patients who refused surgery were given bevacizumab (5 mg/kg IV) combined with TMZ (150 mg/m^2^/d orally for 5 days, repeated every 21 days for 6 cycles), followed by bevacizumab (3 mg/kg) and tislelizumab (200 mg IV) every 21 days for six cycles.

### Sample collection, DNA extraction, and library preparation

**Fig. 1:**
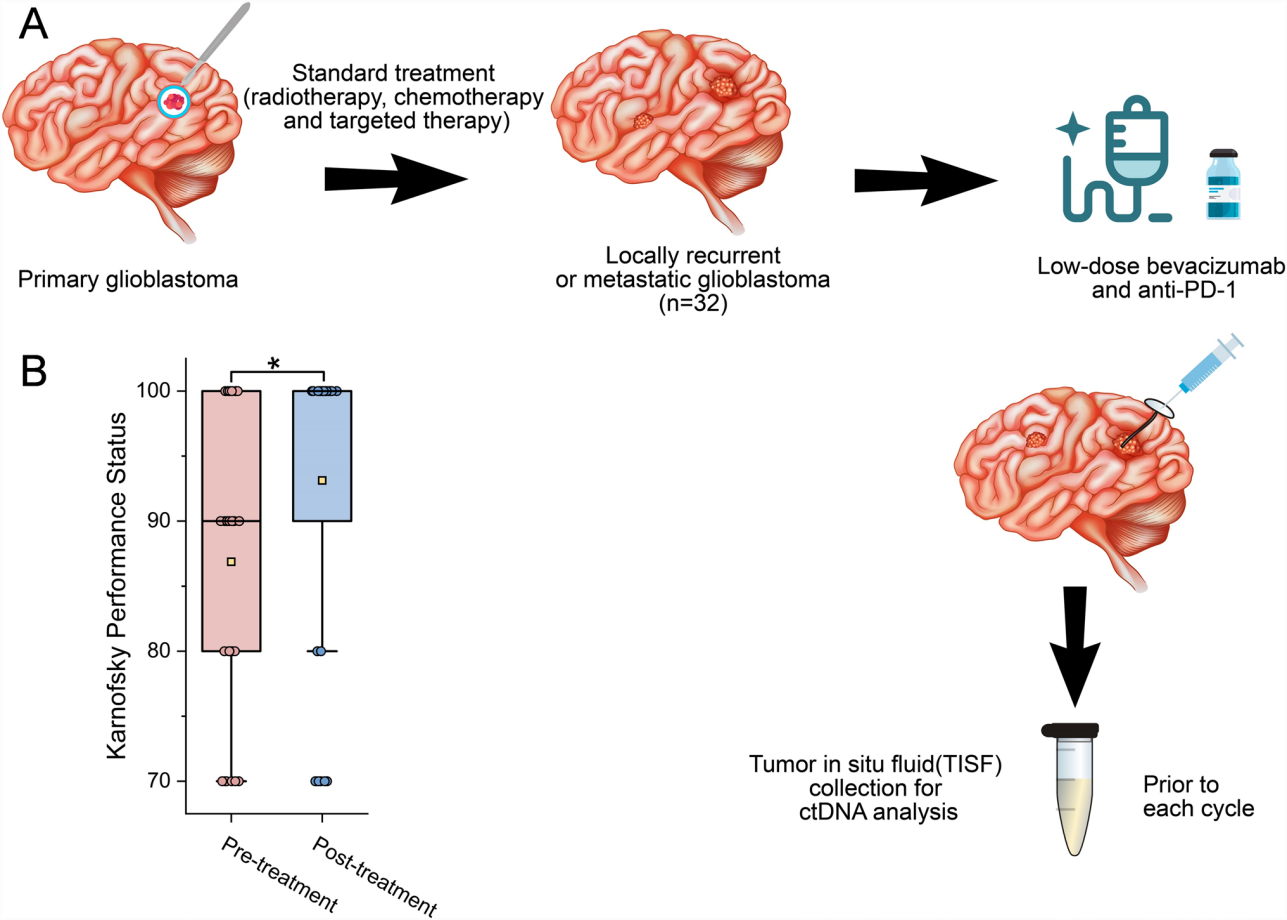
Treatment schema and patient characteristics. **A**, Schematic showing the timing of treatment and tumor in situ fluid (TISF) collection. TISF was collected for ctDNA analysis pre-treatment and prior to each cycle of immunotherapy. **B**, The KPS scores of 32 patients with recurrent glioblastoma treated with anti-PD-1 antibody combined with low-dose bevacizumab were significantly higher than those before treatment

Tumor in situ fluid (TISF) samples were collected as previously described [18–20, 22]. A small amount of TISF (0.5–2 ml) was obtained by syringe from the implanted reservoir sac every 4 to 8 weeks (Fig. 1A). TISF is the fluid present in the local surgical cavity. ctDNA profiles from tumor tissue and TISF samples can be used to assess the dynamic evolution of the tumor in real time, while 5 ml of blood is collected as a germline DNA control.

Genomic DNA (gDNA) and cell-free DNA (cfDNA) were extracted from fresh tissue, formalin-fixed, paraffin-embedded (FFPE) tissue, leukocytes, and TISF using kits (Kai Shuo, Thermo), according to the manufacturer's instructions. DNA was quantified using the Qubit dsDNA HS Assay Kit (Thermo, Fisher) and its quality assessed using the Agilent 4200 TapeStation (Agilent).

### Library sequencing and bioinformatics analysis

Qualified DNA libraries were sequenced using the Illumina NovaSeq6000 platform (Illumina, San Diego, CA) to generate 150 bp paired-end reads. Adapter trimming and filtering of low-quality bases were performed using the software fastp (v.2.20.0). Reads were aligned to the reference genome (hg19, GRCh37 of UCSC) using BWA-MEM (v.0.7.17). Duplicate reads were removed using Dedup and Error Correct. SNVs/indels were called and annotated using VarDict (v.1.5.7) and InterVar, respectively, and screened for common SNPs from public databases (1000 Genome Project, ExAC). CNVs were analyzed using CNVkit (dx1.1) and fusion genes using factera (v1.4.4).

### TMB calculation

To calculate the TMB using the 551-solid cancer-gene targeted next-generation sequencing (NGS) panel, all base substitutions and indels in the coding region of targeted genes were summed, excluding synonymous alterations, alterations with AF < 0.02, and alterations listed as known somatic alterations in COSMIC.

### Statistical analysis

The primary outcome was overall survival (OS, defined as time from enrollment to death or last clinical follow-up). Secondary outcomes included OS rate at 12 months, progression-free survival (PFS, defined as time from treatment initiation to first disease progression, death, or last follow-up imaging), and the objective response rate (ORR, defined as complete response plus partial response). The mutant allele fraction (MAF) was defined as the sum of all mutations detectable in each sample. Exploratory endpoints included drug safety and toxicity (Common Terminology Criteria for Adverse Events, CTCAE 5.0). The functional status of tumor patients was assessed using the Karnofsky Performance Status (KPS) scoring criteria. PFS and OS were analyzed using the Kaplan–Meier method, and the stratified Cox proportional hazards model was employed to calculate the hazard ratio (HR) and 95% confidence interval (CI). Clinical response was assessed using RANO 2.0 criteria, classifying responses as complete response (CR), partial response (PR), stable disease (SD), or progressive disease (PD) [23]. The Wilcoxon rank-sum test compared continuous variables between two groups, while Spearman's rank correlation estimated the correlation between two continuous variables. *P* < 0.05 indicated statistical significance. Statistical analyses were conducted using Prism 9.5 or R, version 4.2.1.

## Results

### Patient characteristics

Between March 28, 2022, and December 31, 2023, 32 rGBM patients were enrolled (Fig. 1A). The median time from diagnosis to relapse was 5.5 months (range, 1.6–10.3 months), with a median age of 52.5 years (range, 49–65 years), and 53.1% (*n* = 17) were female (Table 1). KPS scores were significantly higher after treatment with tislelizumab and low-dose bevacizumab than before treatment (*P* = 0.038, Table 1, Fig. 1B).

**Table 1:** Summary table of patient characteristics

Characteristic	Median (range) or number (%)
Age, median (range), y	52.5(49–65)
*Sex*	
Female	17(53.1)
Male	15(46.9)
*Histology*	
Glioblastoma	32(100.0)
Tislelizumab + low-dose bevacizumab was discontinued	22(68.6)
Disease progression	21(95.5)
Drug-related toxicity	1(4.5)
Radiotherapy completed	18 (56.3)
Temozolomide received	32(100.0)
Reoperation	3(9.4)
*MGMT promoter methylation status*	
Methylated	12(37.5)
Unmethylated	12(37.5)
Not reported	8(25.0)
*Time from initial diagnosis to recurrence*	
Median (range), mo	5.5(1.6–10.4)
< 1 year	23(71.9)
> 1 year	9(28.1)
*Karnofsky Performance Status at study entry*	
100	10(31.3)
90	8(25.0)
80	8(25.0)
70	6 (18.7)
< 70	0
*Karnofsky Performance Status at study entry*	
100	24(75.0)
90	0
80	2(6.3)
70	6(18.7)
< 70	0

At the data cutoff (December 31, 2023), with a median follow-up of 11.0 months (95% CI, 9.0–16.2), 22 patients (68.8%) had discontinued study treatment, primarily due to disease progression (*n* = 21, 95.5%) and study drug-related toxic effects (*n* = 1, 4.5%) (Table 1). All patients received at least one cycle of combination therapy, with a median of 4.5 cycles completed, allowing efficacy evaluation using RANO 2.0 criteria.

### Circulating tumor DNA analysis

**Fig. 2:**
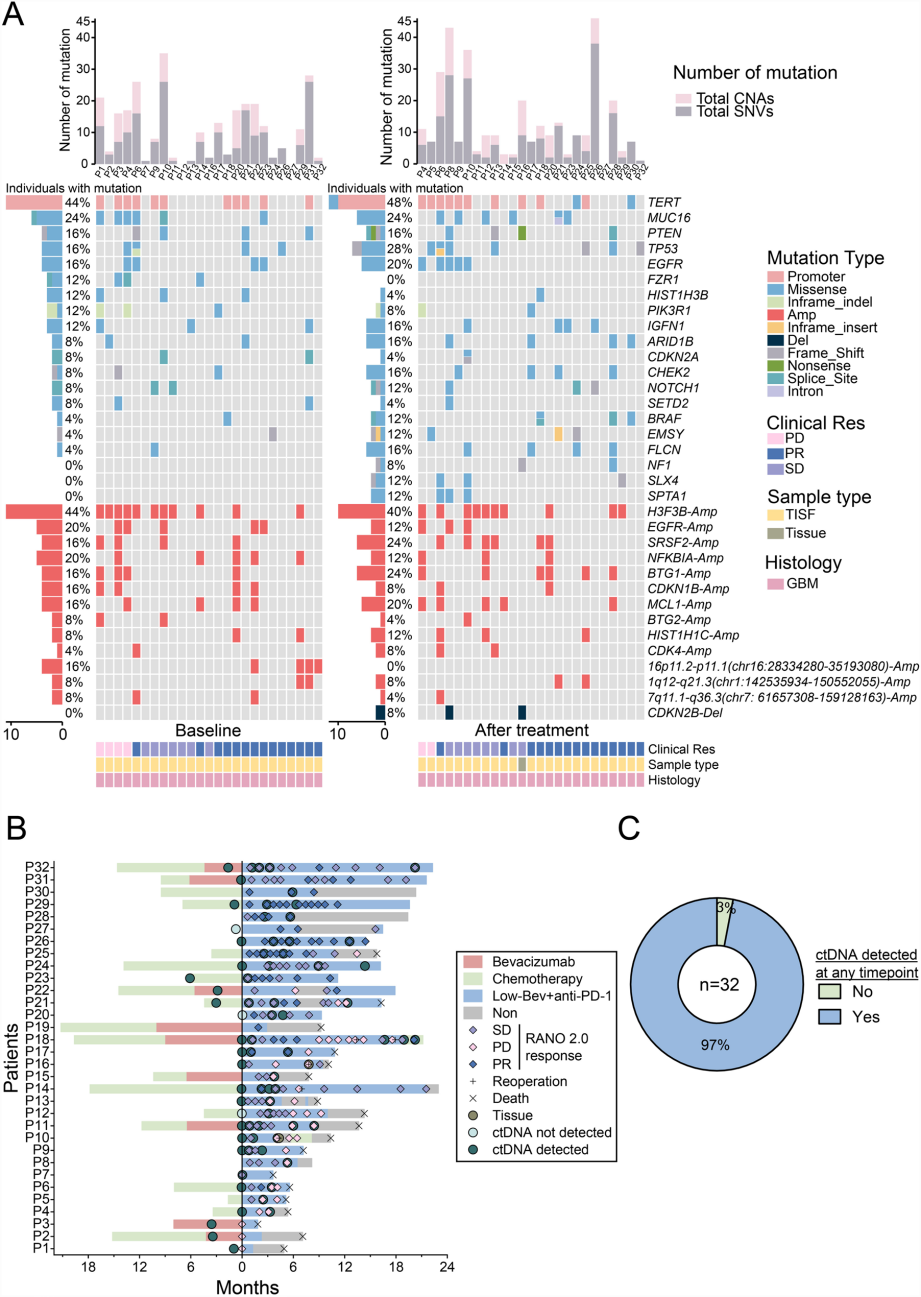
Patient treatment events and ctDNA outcomes. **A**, Oncoplot depicting the genomic alteration of 32 recurrent GBM patients at different time points. Plot of tumor variants identified from 551-panel sequencing and tracked using ctDNA analysis for each patient. The top panel shows the total number of single nucleotide variants (SNVs) and copy number alterations (CNAs) tracked, and the left panel shows the number of patients with mutations in each gene. Only the most frequently mutated genes are displayed. **B**, Event chart showing time points for low-dose Bev + anti-PD-1 treatment, treatment response assessed according to RANO2.0 criteria, and the results of ctDNA testing for each patient with at least one TISF sample or tissue-sample time point analyzed. **C**, Proportion of patients with ctDNA detected in at least one TISF sample time point. Treatment efficacy (*PD* progressive disease; *PR* partial response; *SD* stable disease)

Despite challenges posed by the COVID-19 pandemic, at least one TISF or tissue sample from 31 patients was analyzed by ctDNA, with 19 samples containing before-and-after controls. High-throughput sequencing of TISF using a custom panel designed for solid tumors was performed (Fig. 2A). TERT emerged as the most prevalent genetic mutation, consistent with previous studies [24].

**Fig. 3:**
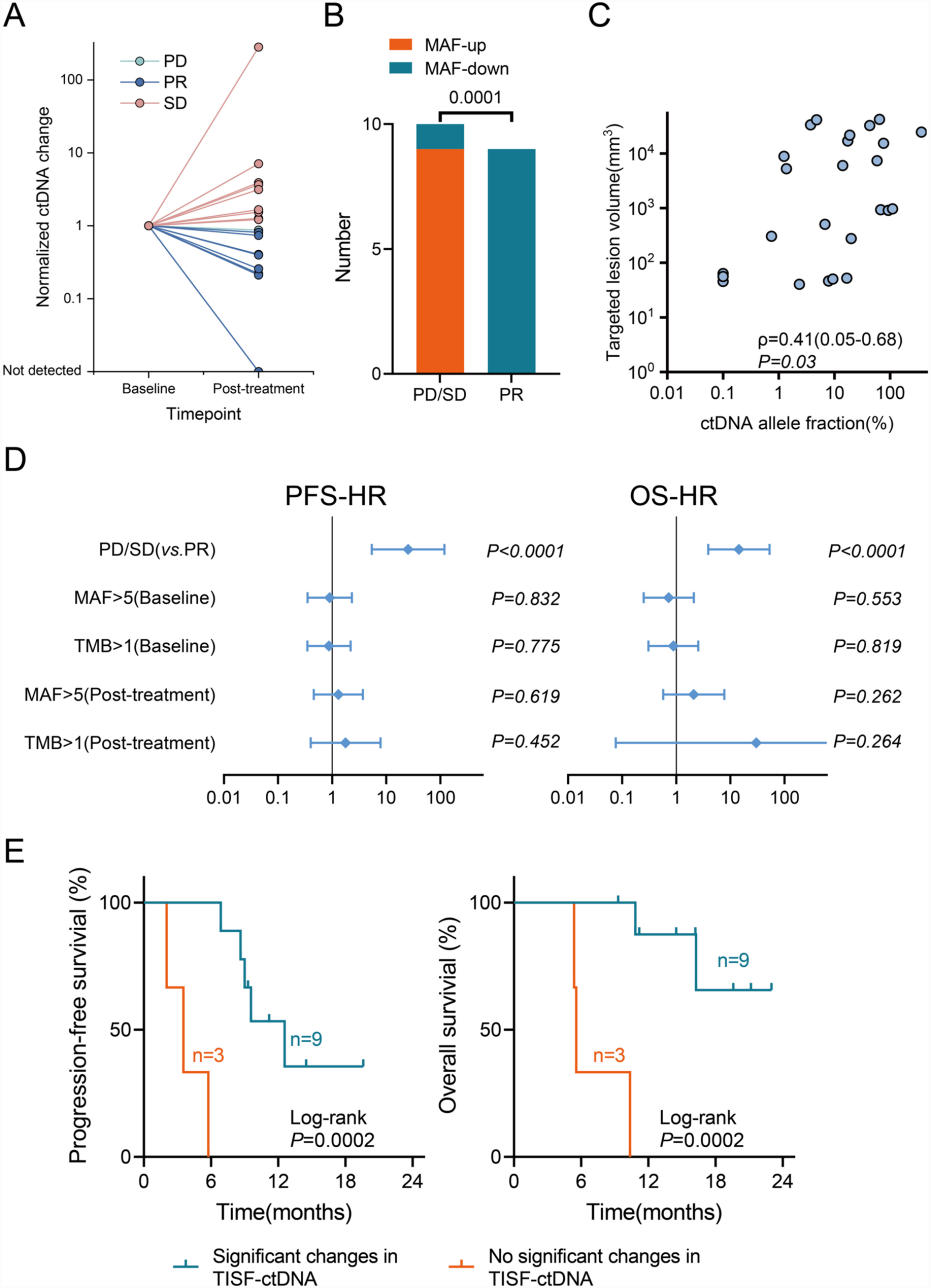
Analysis of ctDNA in patients treated with low-dose Bev + anti-PD-1. **A**, Spider plot of ctDNA levels before and after treatment with low-dose Bev + anti-PD-1 treatment. Patients are colored by RANO2.0 response, and the ctDNA allele fraction at each time point was divided by the pre-treatment allele fraction. In the one patient with ctDNA not detected prior to treatment, the pre-treatment limit of detection was used for normalization based on the number of mutations tracked and average sequencing depth as described in the methods. **B**, Fisher precise test analysis showed that changes in ctDNA levels were significantly correlated with treatment response. **C**, Correlation between baseline tumor burden measured by 3D slicer and baseline ctDNA mutant allele fraction (MAF). **D**, Forest plot depicting progression-free survival (PFS) and overall survival (OS) improvements for each variable in patients treated with low-dose Bev + anti-PD-1 therapy. The HRs and statistical significance of the difference were computed using the Cox proportional hazards model and Wald test. **E**, Kaplan–Meier curves showed that patients in the TISF-ctDNA significant changes group had significantly improved PFS and OS after receiving low-dose Bev plus anti-PD-1 therapy. Spearman's correlation coefficient, 95% confidence interval, and *P*-value are displayed on the graph

To explore ctDNA levels and tumor mutational burden's prognostic predictive value, we calculated the mutant allele fraction (MAF) and tumor mutation burden (TMB) for all detectable mutations in each sample. Baseline ctDNA was detected in 78% of patients (*n* = 25), and ctDNA was detected at least once in 97% of patients (*n* = 31) (Fig. 2B and C). TISF or tissue samples were collected from 19 patients before and after treatment, with ctDNA levels elevated in 9 SD patients and decreased in 10 patients (1 PD patient, 9 PR patients) (Fig. 3A). TISF-ctDNA dynamic changes significantly correlated with treatment response (*P* = 0.0001, Fig. 3A, B). There was a significant correlation between baseline ctDNA levels and tumor volume burden measured on imaging (*P* = 0.03, Fig. 3C). The COX risk regression model showed that RANO 2.0 response assessment (PR *vs*. PD/SD) was significantly associated with PFS and OS (PFS: *P* < 0.0001, *HR*: 25.3; OS: *P* < 0.0001, *HR*: 14.4), while MAF and TMB of ctDNA at baseline and post-treatment did not significantly correlate with PFS and OS (Fig. 3D). Interestingly, 2 patients with ctDNA negative (ctDNA^-^) before or after treatment had better prognosis (P12: PFS 6.0 months, OS 14.3 months; P27: PFS 15.6 months, OS 16.5 months). Among the 12 patients with high baseline TISF-ctDNA levels (MAF > 5%), prognosis improved when gene mutations in TISF-ctDNA significantly changed post-combination therapy (PFS: *P* = 0.0002, *HR*: 0.12; OS: *P* = 0.0002, *HR*: 0.08, Fig. 3E).

**Fig. 4:**
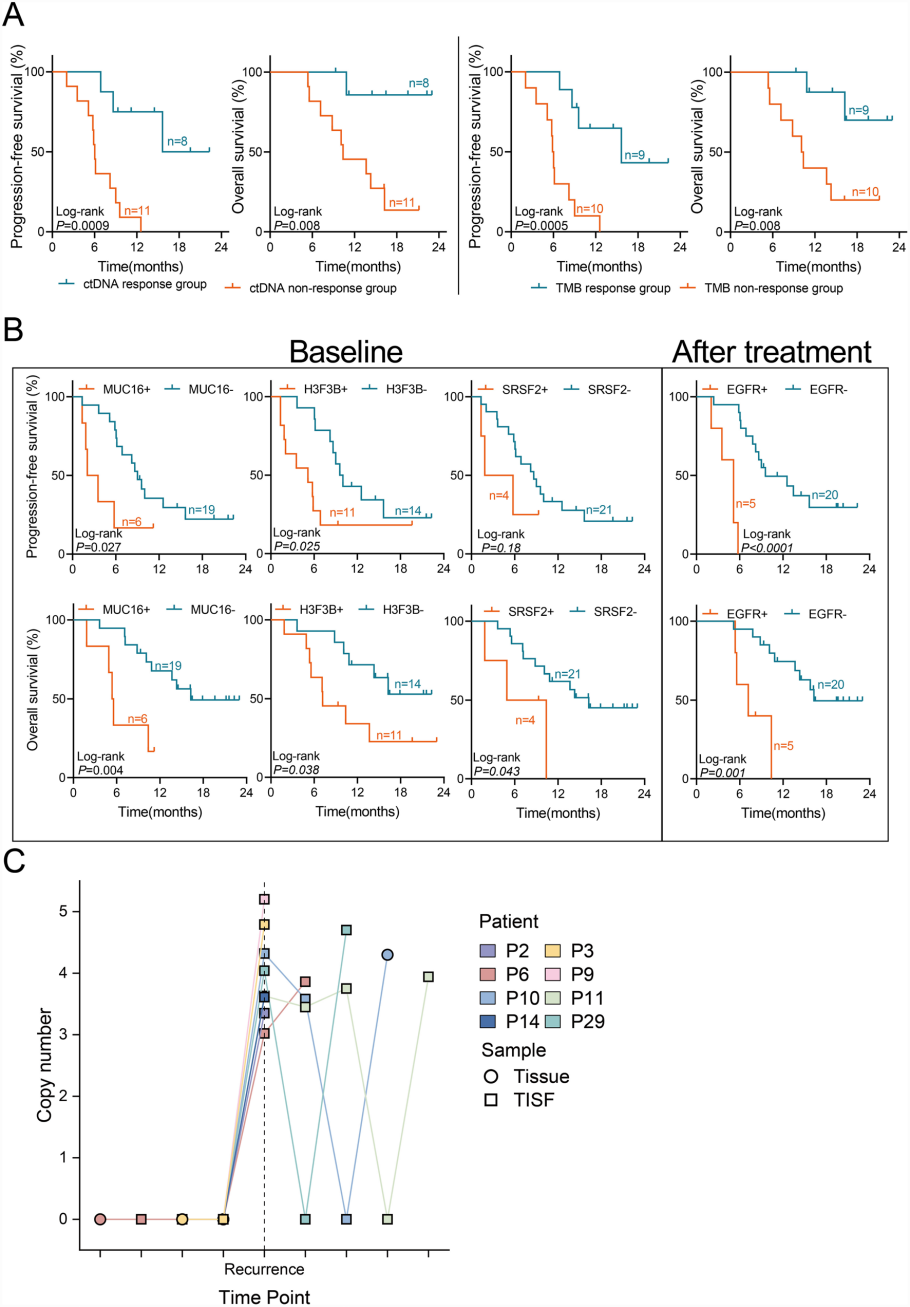
Oncogenic alterations correlated with fewer benefits from low-dose Bev + anti-PD-1 therapy. **A**, Kaplan–Meier curves showed that patients in the ctDNA response group had significantly improved PFS and OS after receiving low-dose Bev plus anti-PD-1 therapy. **B**, Kaplan–Meier curves depict PFS and OS improvements in patients with partial gene wild-type mutations on low-dose Bev + anti-PD-1 therapy. **C**, Copy number changes in H3F3B amplification in 8 rGBM patients throughout treatment

We also analyzed whether MAF and TMB changes could predict prognosis. Patients were divided into ctDNA/TMB response and non-response groups based on whether post-treatment MAF and TMB decreased by 20% compared to baseline. Post-grouping analysis revealed that patients in the ctDNA/TMB response group (≥ 20%) had significantly better PFS and OS (ctDNA: PFS *P* = 0.0009, *HR* = 0.16; OS *P* = 0.008, *HR* = 0.10; TMB: PFS *P* = 0.0005, *HR* = 0.18, OS *P* = 0.008, *HR* = 0.17; Fig. 4A).

### Oncogenic alterations correlated with fewer benefits from low-dose Bev + anti-PD-1 therapy

We applied COX regression models to evaluate whether gene mutations were associated with low-dose Bev + anti-PD-1 efficacy. Stratified analysis of baseline TISF-ctDNA revealed that MUC16 mutation (PFS: *P* = 0.03, *HR* = 2.90; OS: *P* = 0.004, *HR* = 4.20), H3F3B amplification (PFS: *P* = 0.025, *HR* = 3.38; OS: *P* = 0.038, *HR* = 2.87), and SRSF2 amplification (PFS: *P* = 0.18, *HR* = 2.25; OS: *P* = 0.043, *HR* = 3.37) were significantly associated with worse prognosis (Fig. 4B). Post-combination therapy TISF-ctDNA showed only EGFR mutations and amplification significantly associated with poorer OS and PFS (PFS: *P* < 0.0001, *HR* = 7.64; OS: *P* = 0.001, *HR* = 5.41; Fig. 4B). Interestingly, none of the eight samples with primary tumor tissue or pre-recurrent TISF had detectable H3F3B amplification (Fig. 4C), suggesting H3F3B amplification emerged during the period of standard therapy and was associated with resistance and relapse.

**Fig. 5:**
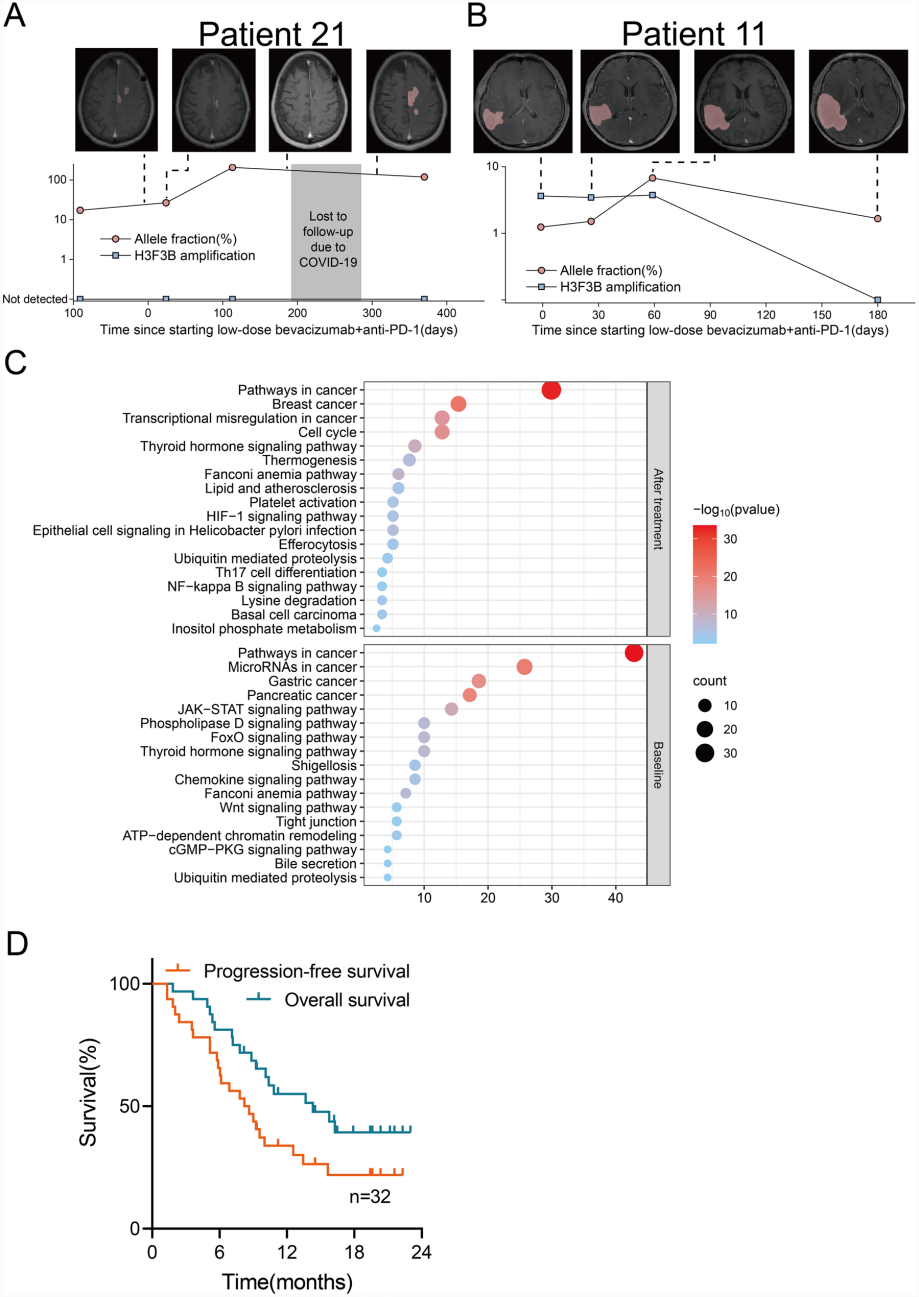
Monitoring response to low-dose Bev + anti-PD-1 therapy using ctDNA analysis. **A**–**B**, Examples of longitudinal radiographic imaging and ctDNA monitoring in **A** a patient with progressive disease on first surveillance imaging and (**B**) a patient with sustained disease remission after starting treatment. Circulating tumor DNA allele fraction is shown in teal, and H3F3B copy numbers are shown in red. **C**, Bubble plots show pathway alterations by KEGG enrichment analysis at baseline and after low-dose Bev plus anti-PD-1 treatment. **D**, Bubble plots showing pathway alterations by KEGG enrichment analysis at baseline and after low-dose Bev plus anti-PD-1 treatment. There was a significant increase in mutated genes associated with cell cycle and transcription dysregulation pathways and a decrease in mutated genes associated with microRNAs in cancer pathways in TISF-ctDNA at relapse compared with baseline. **E**, The median PFS and OS of all patients were analyzed

Two patients demonstrated H3F3B amplification's ability to track combination therapy response. Patient 21 had a near-complete imaging response at 6.4 months but progressed after being lost to follow-up for 3.1 months due to the COVID-19 pandemic (Fig. 5A). Patient 11 consistently had H3F3B amplification detected during follow-up and progressed 1.7 months after starting therapy (Fig. 5B). These results suggest H3F3B amplification may lead to drug resistance by altering the tumor immune microenvironment, and ctDNA has potential to monitor combination therapy response in rGBM patients.

Finally, 8 patients experienced a second relapse after combination therapy, all from the ctDNA/TMB non-response group. KEGG pathway enrichment analysis revealed significant increases in mutated genes associated with cell cycle and transcriptional misregulation pathways in ctDNA at second recurrence and significant decreases in genes associated with microRNAs in cancer pathways (Fig. 5C). More cohort studies are needed to verify these changes in detail, revealing the related mechanisms of low-dose Bev + anti-PD-1 therapy and acquired resistance.

### Patient outcomes and safety

Among all patients, 18 (56.3%) had PR, 9 (28.1%) had SD, and 5 (15.6%) had PD, with an ORR of 56.3%. The 12-month OS was 43.8%. Patients who achieved PR had a median response duration of 13.4 months (95% CI, 7.0–19.9). Median PFS and OS were 8.2 months (95% CI, 5.2–11.1) and 14.3 months (95% CI, 6.5–22.1), respectively (Fig. 5D).

Observed toxicities included anemia (50.0%), fatigue (34.1%), hypokalemia (31.3%), increased alanine aminotransferase (31.3%), and decreased white blood cell count (25.0%). One patient experienced grade 4 acute pancreatitis, and another had tertiary toxicity with elevated ALT levels. No grade 5 adverse events occurred (Table 2).

**Table 2:** Treatment-related adverse events in the safety population

Patients	Immune checkpoint inhibition plus low-dose bevacizumab (*n* = 32)	
	Any grade (%)	Grade ≥ 3
Any treatment-related adverse events	29(90.6)	2(6.2%)
*Treatment-related adverse events occurring in patients*		
Anemia	16(50.0)	0
Fatigue	11(34.4)	0
Increased alanine aminotransferase	10(31.3)	1(3.1%)
Hypokalemia	10(31.3)	0
Decreased white blood cell count	8(25.0)	0
Decreased platelet count	5(15.6)	0
Increased blood creatinine	4(12.5)	0
Nausea	3(9.4)	0
Pneumonia	1(3.1)	0
Pancreatitis	1(3.1)	1(3.1%)
Hypothyroidism	1(3.1)	0

## Discussion

In this study, 32 rGBM patients received tislelizumab plus low-dose Bev, hypothesizing that low-dose bevacizumab would normalize vascular conditions and facilitate immunotherapy, using TISF-ctDNA as a biomarker to track treatment response and gene evolution [8, 9]. The observed ORR of 56.3% significantly benefited patients, exceeding the 7.8% ORR for GBM with nivolumab in the CheckMate 143 trial [13]. This study is the first to perform biomarker analysis in rGBM patients treated with this combination therapy.

Standard-dose bevacizumab combined with anti-PD-1 has been confirmed effective in other solid tumors [28–31], but efficacy in rGBM is poor [32]. Bevacizumab, a humanized monoclonal antibody inhibiting VEGF, enhances tumor-specific immune response by promoting immunosuppressive tumor microenvironment, normalizing vascular structure, increasing T cell infiltration, and activating local immune microenvironment [12, 33–36]. The 2021 ASCO Annual Meeting reported no benefit of low-dose Bev + anti-PD-1 compared with standard Bev for rGBM, and standard Bev can help older rather than younger patients [32]. Therefore, it is essential to find suitable biomarkers that guarantee to maximize the therapeutic effect [37]. However, TMB and PD-L1 expression has not predicted anti-PD-1 monotherapy efficacy in rGBM [38–41]. Although single time-point MAF and TMB expression did not correlate with response to low-dose Bev plus anti-PD-1, dynamic changes predicted response. Two patients with ctDNA negative (ctDNA^-^) before or after treatment had better prognosis, and patients with high baseline ctDNA levels (MAF > 5%) had better prognosis with significant post-treatment TISF-ctDNA gene mutation changes. This facilitates screening high-risk recurrence patients and timely treatment regimen adjustments.

CtDNA is a promising biomarker in solid tumors (lung, breast, prostate, colorectal, melanoma, glioma) [17, 42, 43], used for early cancer detection, treatment selection, MRD detection, recurrence surveillance, and treatment response monitoring [44], used for early cancer detection, treatment selection, MRD detection, recurrence surveillance, and treatment response monitoring [44, 45]. Based on this research, early treatment of high-risk postoperative recurrence GBM patients (i.e., ctDNA recurrence) is planned.

Specific oncogenic alterations can disrupt the cancer immune cycle and influence immunotherapy efficacy [46, 47]. We identified various oncogenic alterations posing higher risk and reducing low-dose Bev + anti-PD-1 therapy benefits, including MUC16 mutation, EGFR mutation, H3F3B amplification, and SRSF2 amplification. MUC16 mutations confer immune evasion and resistance to immunotherapy in tumors [25, 48, 49]. SRSF2 expression correlates with cancer progression in malignant ovarian tissues [27]. Histone H3.3 point mutations are frequently observed in pediatric high-grade glioma (pHGG) [50–53]. But associated copy number variation in glioblastomas has not been reported. Amplification of H3F3B associated with aortic dissection disease may explain resistance to low-dose bevacizumab + anti-PD-1 treatment [26, 54]. EGFR mutation and amplification are poor prognostic markers for glioma [4]. EGFR signaling pathway plays crucial roles in cancer immune evasion [55], with SEC61G as an EGFR-coamplified gene promoting GBM immune evasion [56]. Even with low-dose Bev combined with anti-PD-1, these oncogenic alterations hinder immunotherapy effectiveness. MUC16 mutations, EGFR mutation, SRSF2 amplification, and H3F3B amplification accounted for 24, 20, 12, and 40% of patients, respectively, providing practical value for patient selection.

This study's limitations include the limited data size and all participants were Chinese. Future genomic data from cohorts with low-dose bevacizumab plus anti-PD-1 therapy are needed to validate identified biomarkers. Validation using a combination therapy dataset could demonstrate intrinsic associations between biomarkers and antitumor immunity, affirming their predictive value for immunostimulatory chemotherapy and anti-PD-1 therapy benefits. Technological advancements are needed to reduce genome sequencing costs and ensure speedy analysis for clinical application.

By performing high-throughput sequencing on samples from 97% of patients, we identified four oncogenic risk alterations as reliable biomarkers for low-dose bevacizumab plus anti-PD-1 therapy outcomes in rGBM patients. These findings provide a basis for individualized treatment and future biological studies of its immuno-oncology characteristics, inspiring biomarker exploration of low-dose bevacizumab + anti-PD-1 in other cancer types.

## Conclusions

Anti-PD-1 antibody combined with low-dose bevacizumab can significantly prolong PFS and OS in rGBM patients without significant adverse reactions, improving quality of life and providing a new effective treatment for rGBM. TISF-ctDNA dynamic changes can predict the treatment response, identify drug resistance mechanisms, monitor high-risk recurrence (ctDNA molecular recurrence) populations, and provide a basis for early intervention decision making. TISF-ctDNA characterizes in vivo gene evolution in rGBM patients treated with anti-PD-1 antibody combined with low-dose bevacizumab, providing molecular information for drug resistance mechanism studies in rGBM.

## Data Availability

The datasets used or analyzed during the current study are available from the corresponding author on reasonable request.
